# Exploring the role of mast cells in the progression of liver disease

**DOI:** 10.3389/fphys.2022.964887

**Published:** 2022-09-13

**Authors:** Shizhuan Huang, Haotian Wu, Feng Luo, Bin Zhang, Tianwei Li, Zongrui Yang, Bixuan Ren, Wenze Yin, Dehai Wu, Sheng Tai

**Affiliations:** Department of Hepatic Surgery, Second Affiliated Hospital of Harbin Medical University, Harbin, China

**Keywords:** mast cells, liver disease progression, inflammatory mediators, HLA-G, immunotherapy

## Abstract

In addition to being associated with allergic diseases, parasites, bacteria, and venoms, a growing body of research indicates that mast cells and their mediators can regulate liver disease progression. When mast cells are activated, they degranulate and release many mediators, such as histamine, tryptase, chymase, transforming growth factor-β1 (TGF-β1), tumor necrosis factor–α(TNF-α), interleukins cytokines, and other substances that mediate the progression of liver disease. This article reviews the role of mast cells and their secretory mediators in developing hepatitis, cirrhosis and hepatocellular carcinoma (HCC) and their essential role in immunotherapy. Targeting MC infiltration may be a novel therapeutic option for improving liver disease progression.

## Introduction

Mast cells are tissue-resident hematopoietic cells first observed by Paul Ehrlich in 1877. Mast cells (MCs) are lineages of cells derived from bone marrow precursors in the bone marrow that express and maintain c-kit expression throughout development (Ribatti and Crivellato, 2014). The c-kit ligand is required to amplify the MC population signal from stem cell factor (SCF). However, studies have shown that other actors, such as interleukin-3(IL-3) and nerve growth factor (NGF), can also promote MC growth *in vitro* (Ihle et al., 1983; Matsuda et al., 1991). The lack of granules in circularly oriented MCs indicated that the egress of MCs from the bone marrow was incompletely differentiated. Besides, once they enter body tissues, the cells mature (Jamur et al., 2005; Ribatti and Crivellato, 2014). Mast cells are generally divided into connective tissue type and mucosal type. In mice, they are known as connective tissue mast cells (CTMCs) and mucosal mast cells (MMCs). In humans, mast cells containing both tryptase and chymase (MC_TC_) correspond to mouse CTMCs, while mast cells containing tryptase but without chymase (MC_T_) correspond to mouse MMCs(Ribatti and Crivellato, 2014; Valent et al., 2020). As local tissue sentinels, mast cells play a critical role in host defense against certain parasites, bacteria, venoms, and allergic and other inflammatory diseases (Beaven, 2009; Kolkhir et al., 2021). Activation of mast cells can contribute to metabolic conditions and disease progression by triggering many different pathways, degranulating, and releasing many mediators.

The liver is the only solid organ that uses regenerative mechanisms to ensure that its liver to body weight ratio is maintained at 100% to maintain homeostasis (Michalopoulos and Bhushan, 2021). Mast cells are “allergy-mediated” cells that regulate histaminergic responses when activated. However, it is now increasingly recognized that mast cells are involved in a broader range of pathological activities, including liver disease (Boyce, 2003; Bachelet et al., 2006; Beaven, 2009). This paper reviews the recent studies on the role of mast cells and their secretory mediators in different stages of liver diseases and expounds on the critical role of mast cells in immunotherapy.

## Mechanisms of mast cells activation

**FIGURE 1:**
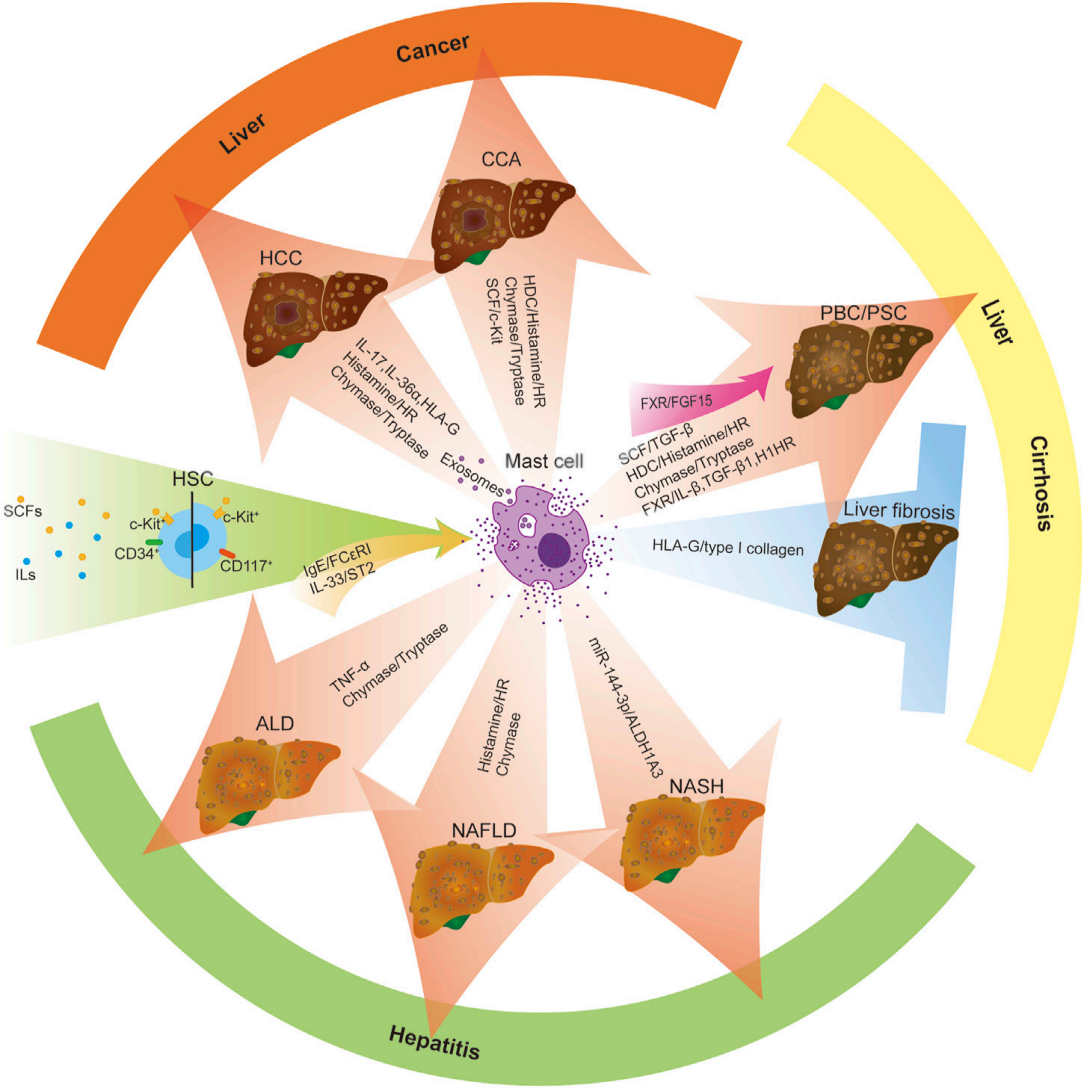
Increased activation and infiltration of MCs regulate liver disease progression. Mast cells are derived from CD34+/CD117+ hematopoietic stem cells stimulated by various stem cell factors (SCFs) and interleukins (ILs). MC activation is triggered by two central receptor-dependent receptor pathways: IgE/FcεRI and IL-33/ST2. Upon activation of mast cells, mast cells reach peripheral organs and develop into mature forms. The presence/infiltration of hepatic mast cells increased, and they degranulated and released many mediators, such as histamine, tryptase, chymase, TGF-β, TNF-α and ILs, bFGF, cytokines, etc., which can modulate the progression of liver diseases (including ALD, NAFLD, NASH, PBC, PSC, HCC, CAA, etc.). Moreover, activated mast cells promote the progression of liver disease by activating MC-mediated signaling pathways, including miR-144-3p/ALDH1A3, HDC/histamine/HR, SCF/TGF-β1, FXR/IL-β/TGF-β 1/H1HR, FXR/FGF15, SCF/c-Kit. Besides, mast cells can also play an anti-fibrotic role in liver disease by secreting HLA-G, which mediates the reduction of type I collagen. Red arrow: promoting factors Blue arrows: inhibitory factors Green Arrows: initiating factors.

Mast cells (MCs), one of the most widely studied innate immune system cells, are individual tissue-resident immune cells from the myeloid lineage. MCs are present throughout our body’s connective tissue, contain coarse granules and potent inflammatory mediators such as histamine, and have long been associated with the pathogenesis of allergies and autoimmune diseases. In addition, mast cells derived from CD34+/CD117+ hematopoietic stem cells stimulated by various stem cell factors (SCFs) and interleukins (ILs) in humans and rodents regulate the progression of liver disease (Ma et al., 2017). The distribution and activation of MC are mediated by IgE/FcεRI and IL-33/ST2 signaling pathways (Turner and Kinet, 1999; Liu et al., 2019; Pham et al., 2021). Upon activation, MCs secrete many peptidases and mediators, including chymase, tryptase, and histamine (HA) (Gruber et al., 1997; Akers et al., 2000; Caughey, 2007). Activated MCs also secrete various pro-inflammatory cytokines, mediating various downstream signaling pathways (Woolley and Tetlow, 2000). After the liver injury, mast cells are activated, the number of mast cells in the liver increases, and the degranulation of mast cells release a variety of mediators such as histamine, heparin, tryptase, chymase, TGF-β1, TNF-α, ILs, cytokines, basic fibroblast growth factor (bFGF) and leukotrienes (LT) B4, LTD4, prostaglandins, etc. (Lappalainen et al., 2004; Galli et al., 2008; Halova et al., 2012; Bulfone-Paus and Bahri, 2015; Komi et al., 2020), which can regulate the progression of liver disease (Figure 1). In the following paragraphs, we review the role of mast cells in regulating liver disease progression at different stages.

## The role of mast cells in hepatitis

### The role of mast cells in NASH

Nonalcoholic fatty liver disease (NAFLD) consists of two different diseases: 1) NAFL, including steatosis or steatosis with mild lobular inflammation; 2) Nonalcoholic steatohepatitis (NASH), including varying degrees of fibrosis, cirrhosis, and HCC(European Association for the Study of the Liver, 2016). Nonalcoholic fatty liver disease (NAFLD) is a simple form of steatosis that can progress to nonalcoholic steatohepatitis (NASH) (Loomba et al., 2021), which is characterized by liver inflammation, fibrosis, and microvesicular steatosis (Powell et al., 2021). MCs can lead to microvesicular steatosis, ductal reaction (DR), biliary senescence, inflammation, angiogenesis, and liver fibrosis during NAFLD/NASH. In wild-type (WT) mice fed a Western diet (WD) and human NASH, the presence of MC was enhanced, and DR increased. Steatosis, DR/biliary senescence, inflammation, liver fibrosis, and angiogenesis were significantly increased in WT WD mice compared to WT CD mice but significantly decreased in Kit^W-sh^ WD mice. Deleting MCs significantly reduced microvesicular steatosis in zone 1 hepatocytes, while injection of MCs into WT and MC-deficient mice promoted WD-induced biliary and liver injury and microcystic steatosis, specifically upregulating microvesicular steatosis in zone 1 hepatocytes (Kennedy et al., 2021). In addition, the aldehyde dehydrogenase 1 family member A3 (ALDH1A3) expression was decreased in WT WD mice and human NASH but increased in Kit^W-sh^WD mice. The expression of miR-144-3p increased in WT WD mice and human NASH but decreased in Kit^W-sh^ WD mice, and the miR-144-3p was found to target ALDH1A3. The results show that MCs drive the deteriorating phenotype through miR-144-3p/ALDH1A3 signal pathway and promote the progression of NAFLD to NASH and microvesicular steatosis (Kennedy et al., 2021) (Figure 1). Inhibiting the activation of MC may be a therapeutic option for NAFLD/NASH.

### The role of mast cells in ALD

Alcohol-related liver disease is the leading cause of liver disease worldwide (Rehm et al., 2009). The densities of tryptase- and chymase-positive mast cells correlated significantly with the degree of fibrosis, and the density of tryptase- and chymase-positive MCs increased in ALD liver biopsies (Matsunaga and Terada, 2000). Mast cells increase in number as hepatic fibrosis progresses, and that mast cells play a role in hepatic fibrosis, probably by secreting fibrogenic substances or by direct secretion of extracellular matrix proteins (Matsunaga et al., 1999). In the pathogenesis of Alcoholic Hepatitis (AH), TNF-α has emerged as a critical factor in the inflammatory process. Data obtained from animal experiments have demonstrated that TNF-α exerts vascular effects by increasing vascular permeability and causing vasodilation. In addition, the study showed that the presence of mast cells and the expression of inflammatory markers such as NF-κB, cyclooxygenase-2, TNF-α, and IL-6 were significantly increased in the liver of ethanol-treated rats. These parameters, including the presence of mast cells, were reduced in ethanol-fed rats treated with zingerone (Mani et al., 2016). Zingerone may be a therapeutic strategy for protecting from ethanol-induced hepatotoxicity through its antioxidant and anti-inflammatory effects.

### The role of mast cells in viral hepatitis

Hepatitis C virus (HCV) remains one of the leading causes of chronic viral infections worldwide, and hepatic steatosis is a common pathological manifestation in patients with chronic HCV-related diseases (Castera et al., 2003; Castera et al., 2004; Hezode et al., 2004; Negro, 2004; Castera et al., 2005; Patel et al., 2005). The study showed a statistically significant difference in MC density between HCV-infected patients with and without steatosis. It is the first study to show a significant increase in the density of MC extracted from tissues of patients affected by chronic HCV infection and histologically documented steatosis (Franceschini et al., 2007). In the study of Koruk, S.T. et al., the accumulation of MCs in chronic HCV patients could serve as an indicator of pathogenesis. They observed that fibrosis and steatosis in the portal venous region increased with the increase and accumulation of MCs, which may serve as follow-up indicators for these patients (Koruk et al., 2011).

## The role of mast cells in liver cirrhosis

### The role of mast cells in NAFLD, liver fibrosis

Nonalcoholic fatty liver disease (NAFLD) is prevalent comorbidity of obesity, which can progress from simple steatosis to nonalcoholic steatohepatitis, cirrhosis, and hepatocellular carcinoma (HCC) (Loomba et al., 2021; Powell et al., 2021). The progression of NAFLD to NASH was facilitated by upregulating acetaldehyde dehydrogenase 1 family, member A3 (ALDH1A3), and down-regulating microRNA-144-3 primer (miR-144-3p) in human NASH liver and wild type (WT) mice fed a western diet (WD). The NAFLD phenotype was ameliorated and shifted towards macrovesicular steatosis in WD-fed MC-deficient, Kit^W-sh^ mice (Kennedy et al., 2021). The accumulation of mast cells in sites such as the portal vein area or the fibrous septum contributed to the progression of fibrosis. There was a strong positive correlation between the number of mast cells, especially those located in the portal vein and fibrous septum, and the degree of liver fibrosis (r = 0.736, *p* < 0.0001), indicating that mast cells were involved in the pathogenesis of liver fibrosis and might play a central role in the pathogenesis of liver fibrosis in patients with nonalcoholic fatty liver (Lewandowska et al., 2020).

In the article, the authors studied the distribution of mast cells in normal human liver and human nonfibrotic and fibrotic liver disease as well as in normal rat liver and acutely and chronically injured rat liver (Armbrust et al., 1997). In normal human and rat livers, mast cells were rarely found in portal tracts, and there was no change in cell numbers in nonfibrotic human or acutely injured rat livers. In contrast, cirrhotic human and rat livers contained numerous mast cells in the portal tracts and the fibrous septa (Armbrust et al., 1997). Therefore, the study showed that mast cells participated in the development of liver fibrosis.

Furthermore, as revealed by immunoperoxidase staining, liver and rat peritoneal mast cells exhibited strong immunoreactivity against the serpins alpha 1-antitrypsin (AAT), alpha 1-antichymotrypsin (ACT) and antithrombin III (AT III). Studies of serial sections identified serpin-positive cells at the same location as metachromatic dyes, indicating that these serpin-positive cells are mast cells. Besides, normal and acutely injured rat liver contained only a few serpin-positive cells exclusively located near vessels of the portal tracts. In contrast, cirrhotic livers contained numerous serpin-positive cells. The results indicate that mast cells may inhibit matrix degradation by displaying protease inhibitors in the late stages of liver fibrogenesis. (Armbrust et al., 1997).

### The role of mast cells in cholestatic cirrhosis

Mast cells (MCs) infiltrated the liver and promoted ductal reaction (DR), biliary hyperplasia, vascular cell activation, biliary senescence, and liver fibrosis during cholestasis. Knockout of MCs reduced biliary hyperplasia, liver injury, and liver fibrosis caused by bile duct ligation (BDL) (Hargrove et al., 2017). Mast cells are considered the primary source of histamine and play a key role in allergy as a pro-angiogenic factor, releasing a large amount of histamine after activation. Mast cells also secrete various factors, including VEGF (Gonzalez et al., 2022). In experiments on the isolation and identification of MCs from the cholestatic liver, mast cells infiltrated the liver after BDL. Biliary tract proliferation, expression of IL-10 and TGF- β 1, and liver fibrosis were significantly increased in BDL rats treated with NaCl MC supernatant, while the infiltration and histamine release of mast cells and the parameters mentioned above were decreased in BDL rats treated with cromolyn Sodium MC supernatant (Hargrove et al., 2016). Furthermore, K. Kyritsi et al. showed that MCs induced DR, biliary tract proliferation, senescence, stellate cell activation, and liver fibrosis through MC-derived TGF-β1, while MCs lacking TGF-β1 reversed the process (Kyritsi et al., 2021). These data strongly suggest that mast cells play a crucial role in regulating biliary proliferation, inflammation, and liver fibrosis.

Primary sclerosing cholangitis (PSC) is characterized by increased biliary tract damage/aging, inflammation, and liver fibrosis. Conclusive shreds of evidence show that MCs are active participants in autoimmune liver diseases such as hepatitis (Francis and Meininger, 2010). Excessive liver fibrosis and collagen deposition can lead to extensive scarring of the liver and ultimately cirrhosis. Cholangiocytes secrete stem cell factor (SCF), a c-kit chemoattractant expressed on MC. Elevated biliary SCF expression and serum SCF levels in human PSC patients induce MC migration and lead to biliary tract injury/liver fibrosis. In Mdr2^−/−^ mice (a primary sclerosing cholangitis (PSC) model), inhibition of SCF by Vivo-Morpholino treatment reduced MC migration, biliary proliferation/and senescence, stellate cell activation, and liver fibrosis (Meadows et al., 2019). Moreover, H1/H2HR and MC were increased in human PSC and CCA, and histamine (HA) increased biliary hyperplasia through H1/H2 histamine receptor (HRs). Liver fibrosis, biliary tract proliferation, collagen deposition, and stellate cell activation were decreased in Mdr2^−/−^ mice treated with H1HR or H2HR inhibition therapy or Sodium Cromolyn treatment (inhibition of histamine secretion) compared with saline treatment (Kennedy et al., 2014; Jones et al., 2016; Kennedy et al., 2018). Besides, Knockout of HDC/histamine signaling in DKO mice improved liver injury, ductal response, liver fibrosis, biliary tract proliferation, inflammation, vascular cell changes, bile acid signaling, and H1HR/PKC-α/TGF-β1 and H2HR/pERK/VEGF-C signaling. However, reactivation of the HDC/HA axis increased these parameters (Kennedy et al., 2020). These data suggest that the HDC/HA axis plays a crucial role in PSC progression and that inhibition of this axis may be a therapeutic tool for patients. In addition, MC infiltration and MC-derived histamine increase biliary tract injury, fibrosis, and inflammation via interaction with histamine receptors following a liver injury such as primary sclerosing cholangitis in mice and humans. Ursodeoxycholic acid (UDCA) treatment inhibited MC activation and reduced the numbers of MC, HDC/histamine/HR axis, HSC activation, inflammation, and fibrosis in Mdr2 mice and PSC patients (Meng et al., 2018). In conclusion, the development of targeting MC therapeutics may offer a new therapeutic opportunity for chronic immune-mediated cholestatic liver disease.

Primary biliary cirrhosis (PBC) is an autoimmune chronic liver disease characterized by progressive bile duct destruction, ultimately leading to cirrhosis, liver failure, and death (Invernizzi et al., 2010). Liver chymase levels were significantly higher in patients with AIH (autoimmune hepatitis) and PBC than in patients with acute hepatitis, and chymase was co-localized with liver fibrosis. Immunoreactive mast cells were detected in the portal vein area and sinus wall, which were consistent with the fibrosis area when chymase immunostaining was performed on sections from patients with AIH and PBC. Therefore, chymase is associated with liver fibrosis in AIH and PBC(Satomura et al., 2003).

The expression of MC and Farnesoid X Receptor (FXR) increased in patients with cholestatic liver disease. The expression of FXR mRNA in the liver of BDL WT mice was significantly increased compared with WT mice, while the expression of FXR in the liver of BDL Kit^W-sh^ mice was lower than that of WT and BDL WT mice. These results indicate that MCs are involved in regulating liver FXR expression. MC-FXR regulates the activation of MC in the liver by activating IL-1 β, TGF- β 1, and H1HR signal pathways, while MCs synergistically regulate the biliary FXR/FGF15 signaling pathway and the expression of IL-1β, TGF-β1 and H1HR by activating FXR (Figure 1). Inhibiting MC-FXR reduces DR, inflammation, biliary senescence, SASP, and liver fibrosis (Meadows et al., 2021).

### The anti-fibrotic effect of HLA + mast cells

The expression of HLA-G protein was confirmed for the first time in cytotrophoblasts at the fetal-maternal interface (Kovats et al., 1990). HLA-G, a member of the HLA- Class IB family, has a membrane-bound and soluble (sHLA-G) form, an immunomodulatory molecule that participates in tumor escape by promoting the Th2 cytokine environment and inhibiting immune effector cells. L. Amiot et al. found that HLA-G was expressed in certain HCV-infected human liver tissue cells and the number of HLA-G positive cells was significantly correlated with the area of fibrosis on tissue sections (Amiot et al., 2014). In addition, they identified the cell type for the first time that expressed HLA-G as mast cells, and HLA-G was expressed by mast cells in liver fibrosis areas of HCV-infected patients. The expression of HLA-G in mast cells was upregulated when mast cells were stimulated by class I interferon (including IFN-α, IFN-β, and IFN-ω) and IL-10 (Amiot et al., 2014).

L. Amiot et al. demonstrated that the anti-fibrosis effect of HLA-G and mast cells was firstly demonstrated in the human liver, and the interaction between HSC and mast cells leads to mast cells attracting and adhering to HSC and significant the reduction of the production of collagen *in vivo* and *in vitro* (Amiot et al., 2019) (Figure 1). This interaction increased the expression and secretion of HLA-G by mast cells. HLA-G acts in an autocrine/paracrine manner by interacting with the receptor ILT2 expressed on stellate cells and mast cells or with stellate cells, thereby reducing collagen production by HSCs(Amiot et al., 2019). Effect of HLA-G on type I collagen degradation: the effect of recombinant HLA-G on HSC/HMC co-culture was studied. The completely soluble HLA-G was co-cultured with HSC/HMC at a concentration of 1μg/ml for 72 h. They found that the addition of exogenous recombinant HLA-G to HSC/HMC co-cultures resulted in a significant reduction in type I collagen levels, approximately half of those in the absence of HLA-G (*p* < 0.01; n = 6) (Amiot et al., 2019). Therefore, Mast cells play a beneficially anti-fibrotic role in liver fibrosis through HLA-G-mediated reduction of type I collagen.

Quantitative expression and properties of HLA-G+ cells in alcoholic fibrosis: N. Mouchet et al. have demonstrated that mast cells can express HLA-G in a basal state, with increased expression in certain cytokine-rich environments, particularly in liver fibrotic tissue. In alcoholic cirrhosis, morphological characteristics of HLA-G+ cells correspond to mast cells, and identification of HLA-G+ cells showed that 51% were mast cells, and mast cells expressing HLA-G were located in the fibrosis region (Mouchet et al., 2021). As with liver fibrosis caused by the hepatitis C virus, the authors found that half of the HLA-G+ cells in alcohol-induced cirrhosis were mast cells, and the liver region was significantly reclassified, in which 63-92% of HLA-G+ cells in the fibrosis region were mast cells, while only 3-23% of cell nodes were mast cells (Mouchet et al., 2021). In conclusion, HLA-G+ mast cells exist in the liver fibrosis region, and HLA-G+ mast cells have a protective effect on inflammation and liver fibrosis.

## The role of mast cells in liver cancer

### The role of mast cells in HCC

Hepatocellular carcinoma (HCC) remains a global health challenge and is the second leading cause of cancer-related deaths, with an increasing incidence worldwide (Llovet et al., 2016; Villanueva, 2019; Llovet et al., 2022). MC integration in HCC occurs through regulation of the IL family, histamine and histamine receptors (HRs), tryptase- and chymase-positive MCs, and MC-derived exosomes (Pham et al., 2022). Mast cells can support tumor growth of hepatocellular carcinoma by expanding suppressor cells, resulting in a poor prognosis (Yang et al., 2010; Tu et al., 2016; Hu et al., 2018). Liao, R. et al. Studies have shown that the high expression of IL-17 and IL-17RE in tumors was significantly correlated with poor survival rate (*p* = 0.016 and <0.001, respectively) and increased recurrence rate (all *p* < 0.001) in patients with HCC(Liao et al., 2013). The high expression of IL-17 and IL-17RE in hepatocellular carcinoma may predict poor prognosis of patients with liver cancer (Liao et al., 2013), while decreased expression of interleukin-36α was associated with poor prognosis of patients with liver cancer (Pan et al., 2013). Studies have shown that MC tryptase + mast cells rather than T cells were the predominant (about 54%) IL-17-expressing cells in *in-situ* HCC tissues, and there was a strong positive correlation between the level of IL-17-producing cells in tumors and the density of microvessels in HCC. It is strongly suggested that IL-17-producing cells in tumors may promote the angiogenesis of HCC (Tu et al., 2016). There was a significant negative correlation between intratumoral MCT + mast cell density and the survival of patients (*p* = 0.0023). The authors found that intratumoral IL-17 + cells and intratumoral MCT + mast cells were associated with poorer survival, the latter being independent prognostic factors. The study shows that the therapy of MC tryptase + mast cells may be an effective strategy to control IL-17 infiltration and MC microvessels in tumors (Tu et al., 2016).

Mast cells (MCs) are enriched in the tumor microenvironment and clinical and experimental studies have confirmed that MCs can promote the occurrence and development of tumors (Milek et al., 2016; Yamashita et al., 2017). Mast cell mediators and histamine and histamine receptors may influence the growth of HCC cells, and mast cells and histamine play different roles according to the characteristics of tumor cells (Lampiasi et al., 2007). It has been shown that down-regulation of miR-940 in HCC cells promoted the overexpression of H1HR in HCC cells, which facilitated the growth and metastasis of HCC cells by inducing cell cycle progression, and formation of lamellipodia, production of matrix metalloproteinase 2, and inhibition of apoptosis. In addition, activation of cyclic adenosine monophosphate-dependent protein kinase A was also involved in H1HR-mediated growth and metastasis of HCC cells. However, an H1HR inhibitor, terfenadine, has significantly inhibited tumor growth and metastasis in a xenograft nude mouse model of HCC(Zhao et al., 2020). This experiment indicates that the targeting therapy of H1HR may be a potential drug target for the treatment of HCC. Moreover, HRH3 was significantly upregulated in HCC tissues. Activation of HRH3 promoted the growth and metastasis of HCC cells. At the same time, inhibition of HRH3 inhibited the metastasis of HCC cells, further supporting the functional role of HRH3 in promoting the metastasis of malignant tumors, which indicates that increased HRH3 levels can be a potential prognostic marker for HCC patients (Yu et al., 2019). Besides, HRH3 in HCC inactivates the cAMP/PKA/CREB pathway by down-regulating the expression of cyclin-dependent kinase inhibitor P21 (CDKN1A), promoting the G1-S phase transition, and ultimately promotes the malignant growth of HCC (Zhang et al., 2020).

Hepatitis C virus E2 envelope glycoprotein (HCV-E2) plays an essential role in regulating immune responses (Sabahi et al., 2014) and may modulate the anti-tumor effect of tumor-infiltrating MCs(Xiong et al., 2017). Xiong, L. et al. Have proved that HCV-E2 increased the expression of miR-490 in MC-derived exosomes and receptor HCC cells, thereby decreasing the activity of the EGFR/AKT/ERK1/2 pathway and inhibiting the migration of HCC cells. Furthermore, transfection of antagomiR-490 on MCs reduced miR-490 in MC-derived exosomes and receptor HCC cells and enhanced the migration and invasion of HCC cells (Xiong et al., 2017). This study provides new ideas for the biotherapy of hepatitis C-induced HCC and also discusses the potential of exosomes miR-490 as a therapeutic target *in vivo*.

Both HLA-G mRNA and protein can be detected in the human HCC cell line (Zeng et al., 2013). The HLA-G molecule has recently been produced by mast cells in the livers of patients infected with the hepatitis C virus (HCV) (Amiot et al., 2014). HLA-G can induce local temporary immunosuppression, inhibit autoimmune or infection-related inflammatory responses, and down-regulate immune responses, such as anti-tumor responses (Amiot et al., 2015). The expression of HLA-G in liver tumors or the high-level expression of sHLA-G in HCC patients can free tumor cells from the immune response and inhibit the properties of immune cells such as T8 lymphocytes, NK cells, B cells, and dendritic cells. Similar to other cancers, HLA-G expression is a factor of poor prognosis (Amiot et al., 2015).

### The role of mast cells in CCA

Cholangiocarcinoma (CCA) is a highly lethal adenocarcinoma of the hepatobiliary system, the second most common type of liver cancer after hepatocellular carcinoma and can be divided into intrahepatic, perihilar, and distal (Brindley et al., 2021; Sarcognato et al., 2021; Zori et al., 2021). Studies have shown that patients with primary sclerosing cholangitis (PSC) are at risk for cholangiocarcinoma (CCA) (Chung et al., 2018). MC was significantly increased in CCA human samples compared with non-malignant or normal liver tissue samples (Terada and Matsunaga, 2000; Johnson et al., 2016), and mast cells (MCs) infiltration and HA levels rose during PSC and CCA. Histamine (HA) increased biliary hyperplasia and promoted the growth of tumors via H1/H2 histamine receptors (HRs) (Kennedy et al., 2018). Therefore, tumor growth, serum HA, angiogenesis, and EMT were reduced in Mdr2−/− mice treated with H1/H2HR antagonist (alone or in combination) compared with physiological salt treatment (Kennedy et al., 2018). These conditions can be treated with HR blockers because patients with PSC are at risk for CCA.

Cholangiocytes promote the growth of CCA via the autocrine release of histamine (Francis et al., 2012). Mast cell infiltration induced increased histamine release, and MCs migrated to the CCA tumor microenvironment via c-Kit/stem factor, increasing tumor progression, angiogenesis, EMT switching, and ECM degradation. Therefore, blocking mast cell-derived histamine can reduce mast cell infiltration and proliferation by inhibiting the SCF/c-Kit signaling pathway or treating sodium cromoglycate and sequentially reducing the abovementioned parameters (Johnson et al., 2016).

## The role of mast cells in immunotherapy

Tumor-associated mast cells have been observed in the solid tumor microenvironment of various cancers. Due to their multifaceted nature and their immunomodulatory effects after activation or degranulation, mast cells have been found to have pro-tumor and anti-tumor effects. Interestingly, tumor-associated mast cells are favorable prognostic factors for certain cancers, such as esophageal adenocarcinoma, ovarian cancer, and diffuse large B-cell lymphoma (Chan et al., 2005; Hedstrom et al., 2007; Wang et al., 2013), but they are also associated with poor or mixed prognosis for other cancers, such as liver cancer, gastric cancer, lung cancer, melanoma, and breast cancer (Takanami et al., 2000; Ribatti et al., 2003; Tu et al., 2016; Reddy et al., 2019; Sammarco et al., 2019). Mast cells are increasingly recognized as a biomarker and an essential determinant of cancer treatment response and an under-recognized but promising target for cancer immunotherapy. In consequence, possible therapeutic pathways for mast cells in cancer include c-kit inhibitors, mast cell stabilizers, activators/inhibitors of the FcεR1 signal pathway, antibodies targeting inhibitory receptors and ligands, and TLR agonists, and mast cell-derived mediators regulators, which target mast cells to ameliorate cancer (Lichterman and Reddy, 2021).

Myeloid-derived suppressor cells (MDSCs) are a heterogeneous population of cells that expand during cancer, inflammation, and infection and can remarkably suppress T cell responses (Gabrilovich and Nagaraj, 2009; Saleem and Conrad, 2011). MDSCs ultimately lead to T cell inhibition by releasing small soluble oxidants, impairing T cell antigen recognition and consumption of essential amino acids in the local extracellular environment during tumor progression (Gabrilovich et al., 2012). MDSCs are transported and accumulated in the liver except at tumor sites, thereby inhibiting Kupffer cells, T cells, and anti-tumor immune responses (Ilkovitch and Lopez, 2009; Connolly et al., 2010; Chan et al., 2011), which facilitate the progression of the tumor. Furthermore, histamine enhanced the expression of T cells inhibition markers Arg1 and iNOS in Monocyte-derived MDSCs. Inhibition of T cells by Monocyte-derived MDSCs was increased after exposure to histamine or culture with MCs (Martin et al., 2014). Histamines released by MCs and MCs are critical for MDSC-mediated immune modulation, and the interaction should be considered in therapeutic interventions targeting MDSCs(Martin et al., 2014).

Anti-PD-1 therapy is used as first-line therapy for many cancers, but there is still a lack of resistance mechanisms to this therapy. In addition, reduced expression of HLA-I and CD8+/Granz B + T cell homeostasis was observed in tumor regions co-located with FOXP3+ Tregs and mast cells, which were associated with anti-PD-1 resistance therapy. Anti-PD-1 combined with sunitinib or imatinib can lead to mast cell reduction and complete tumor regression (Somasundaram et al., 2021). Therefore, the results show that mast cell consumption can ameliorate the efficacy of anti-PD-1 therapy.

## Future perspectives

Liver disease is a significant burden of disease and costs worldwide. Currently, viral hepatitis is the leading cause of acute liver disease, while alcohol and viral hepatitis are the leading causes of chronic liver disease (Asrani et al., 2019). With the deepening of research on mast cells, it has been found that mast cells not only play a role in an allergic reaction, inflammation, anti-parasite, and other aspects but also play a crucial role in facilitating the progression of liver diseases, and these immune cells have been identified as the vital regulatory factor of pathogenic processes. Since mast cells are recruited in large numbers during liver injury, a large number of mast cells are activated and secrete protease and mediators, which promote the progression of liver disease. Therefore, targeting MCs for the migration or activation of mast cells may provide a new therapeutic strategy for treating liver disease. However, we do not fully understand the pathogenesis of mast cells in the progression of liver disease, and further experimental studies are still needed to explore the full role of mast cells in liver disease.
